# Biologic Therapy Carries a Very Low Risk of Reactivation in Hepatitis B Surface Antigen-Negative Phase of Hepatitis B

**DOI:** 10.5152/tjg.2022.22196

**Published:** 2023-02-01

**Authors:** İlkay Ergenç, Haluk Tarık Kani, Murat Karabacak, Elif Cömert Özer, Shahin Mehdiyev, Fuad Jafarov, Kerem Yiğit Abacar, Seda Kutluğ Ağaçkıran, Gizem Sevik, Rahmi Aslan, Fatma Alibaz Öner, Nevsun İnanç, Mehmet Pamir Atagündüz, Dilek Seçkin, Yeşim Özen Alahdab, Tülin Ergun, Haner Direskeneli, Özlen Atuğ

**Affiliations:** 1 Division of Gastroenterology, Department of Internal Medicine, Marmara University Faculty of Medicine, İstanbul, Turkey; 2 Division of Rheumatology, Department of Internal Medicine, Marmara University Faculty of Medicine, İstanbul, Turkey; 3 Department of Dermatology, Marmara University Faculty of Medicine, İstanbul, Turkey

**Keywords:** Anti-HBc IgG, anti-TNF, biologic agents, hepatitis B, reactivation

## Abstract

**Background:**

The risk of hepatitis B reactivation in hepatitis B surface antigen-negative phase of hepatitis B virus-infected patients exposed to biologic agents is not clear. We aimed to investigate the reactivation rate in hepatitis B surface antigen-negative phase of hepatitis B virus-infected patients after biologic therapy.

**Methods:**

Patients followed at gastroenterology, rheumatology, and dermatology clinics with a diagnosis of immune-mediated inflammatory diseases were screened. Immune-mediated inflammatory diseases patients exposed to biologic agents with a negative hepatitis B surface antigen and positive hepatitis B core immunoglobulin G antibody were included in the study.

**Results:**

We screened 8266 immune-mediated inflammatory disease patients, and 2484 patients were identified as exposed to biologic agents. Two hundred twenty-one patients were included in the study. The mean age was 54.08 ± 11.69 years, and 115 (52.0%) patients were female. The median number of different biologic subtype use was 1 (range: 1-6). The mean biologic agent exposure time was 55 (range: 2-179) months. One hundred and fifty-two (68.8%) patients used a concomitant immunomodulatory agent, and 84 (38.0%) patients were exposed to corticosteroids during biologic use. No hepatitis B reactivation with a reverse seroconversion of hepatitis B surface antigen positivity was seen. Antiviral prophylaxis for hepatitis B was applied to 48 (21.7%) patients. Hepatitis B virus-DNA was screened in 56 (25.3%) patients prior to the biologic exposure. Two patients without antiviral prophylaxis had hepatitis B virus-DNA reactivation with a negative hepatitis B surface antigen during exposure to the biologic agent.

**Conclusion:**

We found 2 reactivations and no hepatitis B surface antigen seroconversion in our cohort. Antiviral prophylaxis for patients exposed to biologic agents may need to be discussed in more detail.

**Main Points**

- No reverse seroconversion and very low reactivation rate were seen in a relatively large hepatitis B surface antigen-negative phase of hepatitis B virus-infected patients exposed to biologic agents.
- These are real-life data representing up to 6 lines of consecutive biologic exposure in all immune-mediated inflammatory diseases (IMID).
- Hepatitis B surface antigen-negative phase of HBV-infected patients poses very low risk of reactivation for all biologic agents in all IMID-diagnosed patients.

## INTRODUCTION

Biologic agents originate from human genes and work with an impact on the immune system.^1^ These agents are common and effective treatment options for immune-mediated inflammatory diseases (IMID) with an increasing frequency of use in recent years. Despite its treatment success, biologic agents, particularly anti-tumor necrosis factor (anti-TNF) drugs, are associated with an increased risk of active infection or reactivation of latent infections.

Resolved hepatitis B, also known as hepatitis B surface antigen (HBsAg)-negative phase of chronic hepatitis B (CHB) infection, is one of the challenging issues in patients treated with biologic agents. Clinical practice guidelines published by various international societies suggest several different approaches including antiviral prophylaxis, follow-up with HBV DNA titers, or preemptive treatment.^2-5^ Since these guidelines were published, an increasing number of articles support the safety of using biologic agents in HBsAg-negative and hepatitis B core immunoglobulin G antibody (anti-HBc IgG)-positive CHB patients under treatment with biologic agents.^6-9^

In this study, we aimed to investigate the flare and reactivation rates of HBsAg-negative and anti-HBc IgG-positive patients under biologic treatments.

## MATERIAL AND METHODS

We identified patients who were followed at gastroenterology, rheumatology, and dermatology outpatient clinics with a diagnosis of rheumatoid arthritis (RA), spondyloarthropathies, psoriasis, and inflammatory bowel disease described as IMID between 2013 and 2020. Patients with a negative HBsAg and positive anti-HBc IgG prior to the biologic agent exposure were included in the study. Patients under 18 years old, subjects with insufficient data, and lost to follow-up were excluded from the study.

Electronic and hardcopy patient files were reviewed. Age, sex, the indication of biologic treatment, type of the biologic agent, duration of the biologic exposure, HBsAg, anti-HBc IgG, anti-HBs, HBV viral load, hepatitis B prophylaxis status (prophylactic agent and duration of the prophylaxis), immunomodulatory agent use, and steroid use were recorded.

The study was conducted in accordance with the ethical standards of the responsible committee on human experimentation and the Declaration of Helsinki of 1975, as revised in 2008, and the Marmara University ethics committee approved the study protocol (Date: February 5, 2021, Number: 09.2021.24|1). Informed consent was not obtained since the protocol was designed retrospectively.

Our primary outcomes were hepatitis B reactivation (HBr) which is defined as reverse seroconversion of HBsAg and HBV DNA flare with an increase of alanine transaminase at least 3 times of baseline.^10^ The secondary outcome was a logarithmic increase in HBV DNA levels.

## Statistical Analysis

Categorical variables were reported as frequency (%). Continuous variables with a normal distribution were reported as mean ± standard deviation, and variables with abnormal distribution were reported as median (range) values.

## Results

**Figure 1.**
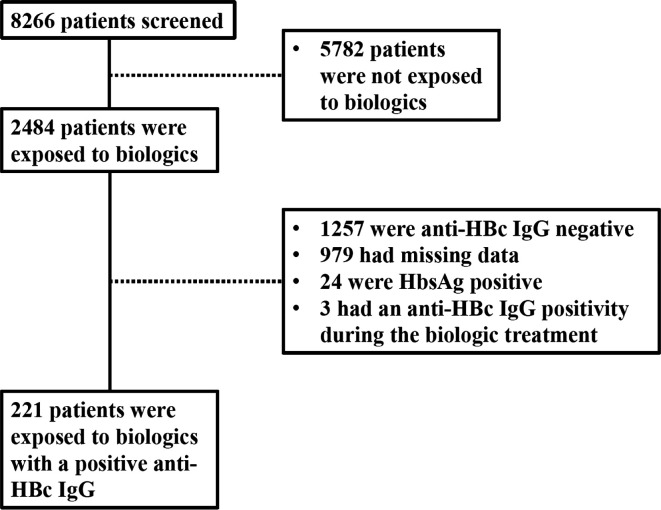
Patients included in the study. Study flow diagram. Anti-HBc IgG, hepatitis B core immunoglobulin G antibody; HBsAg, hepatitis B surface antigen.

A total of 2484 patients were exposed to at least 1 type of biologic treatment. We identified 221 patients with a positive anti-HBc IgG and negative HBsAg (Figure 1). The mean age was 54.08 ± 11.69 years, and 115 (52.0%) patients were female. Patients who were exposed to biologics were diagnosed as follows: 55 (24.9%) had RA, 76 (34.4%) had spondyloarthropathies, 20 (9.0%) had inflammatory bowel disease [4 (20.0%) had e ulcerative colitis and 16 (80.0%) had Crohn’s disease], 57 (25.8%) had psoriasis, 7 (3.2%) had Behcet’s disease, 4 (1.8%) had Takayasu arteritis, 1 (0.5%) had adult-onset Still’s disease, and 1 (0.5%) had giant cell arteritis (Table 1). The median number of different biologic subtype exposure was 1 (range: 1-6) (Table 2). The mean biologic agent exposure time was 55 (range: 2-179) months. One hundred and fifty-two (68.8%) patients used a concomitant immunomodulatory agent, and 84 (38.0%) patients were exposed to corticosteroids during biologic use.

There was no HBr with a reverse seroconversion of HBsAg positivity in the whole cohort. Antiviral prophylaxis for hepatitis B was given to 48 (21.7%) patients as entecavir, tenofovir, or lamivudine. Hepatitis B virus DNA was screened in 56 (25.3%) patients prior to the biologic exposure, and of the 56 patients, HBV DNA was screened in 21 (37.5%) patients during the follow-up. Two patients had HBV DNA positivity with a negative HBsAg during exposure to the biologic agent.

The first case was a 59-year-old male diagnosed with RA in 2008. Since the patient was resistant to methotrexate monotherapy, infliximab therapy was initiated with a dose of 5 mg/kg/every 8 weeks in 2010. Serum transaminase levels were normal, and hepatitis B serology showed HBsAg negative, anti-HBs positive, and anti-HBc IgG positive with a negative HBV DNA. Four years later, HBV DNA level was detected as 20 IU/mL, and prophylaxis with lamivudine was started. On third year of lamivudine prophylaxis, HBV DNA level was detected as 325 IU/L, and the antiviral treatment was switched to tenofovir. Hepatitis B surface antigen reverse seroconversion was not seen. No HBV DNA elevation was seen later on follow-up.

The second case was a 36-year-old man diagnosed with ankylosing spondyloarthritis. After 3 months of non-steroidal anti-inflammatory drug treatment failure, adalimumab 40 mg/every 2 weeks was initiated in 2014 while the patient’s HBV DNA was undetectable. Four years later, on routine tests, HBV DNA level was detected as 35 IU/mL, and entecavir prophylaxis was started. No HBsAg reverse seroconversion or transaminase elevation was seen, and HBV DNA levels were undetected for 3 years under prophylaxis.

## Discussion

Our study demonstrates no HBsAg reverse seroconversion under biologic therapy. Furthermore, we found a very low rate (0.9%) of development of detectable HBV DNA in the absence of reverse seroconversion to positive HBsAg. In addition, no hepatitis B flare was seen in our cohort. Though a single-center study, ours is one of the largest cohorts of HBsAg negative and anti-HBc IgG positive patients exposed to biologic agents.

Anti-TNF agents have been used for several decades and have the highest number of studies when compared to other biologic agents. In a pooled data analysis, HBr was found as 1.7% in patients with rheumatologic diseases exposed to anti-TNF agents.^11^ Also, in another pooled data analysis, which included the case reports, the HBr rate was found as 5%.^12^ Our reactivation rate was lower than both the pooled data analyses. One of the studies only included the rheumatologic diseases, not inflammatory bowel disease or psoriasis, and it may be one of the reasons for the difference between reactivation rates. More importantly, both studies were based on a literature search, and 1 study included the case reports. Therefore, our cohort study reflects the real-life data, and the difference in results may be due to the difference in the design of the studies.

In a retrospective cohort study, there was no HBr in 178 patients treated with anti-TNF agents.^13^ Also, in another retrospective cohort study, HBr was seen in 0.9% of IMID patients exposed to anti-TNF agents.^14^ In patients with RA, HBr was found to be 1.1% in patients exposed to anti-TNF agents and 5.5% in all biologic treatments including anti-TNF agents.^15^ Also, in another RA cohort, HBr was found to be 3.1% in patients who were exposed to anti-TNF agents and 5.2% in all biologic treatments including anti-TNF agents. On the contrary, in another study in patients with rheumatologic diseases, there was no HBr with anti-TNF agents.^16^ In the current literature, reactivation of HBV under biologic agent rates differs between 0% and 5.5%, and we found a 0.9% reactivation rate. Our patient cohort includes all IMID, whereas the studies which found 5.5% and 5.2% reactivation rates were conducted with RA patients. This may be the reason for the difference in the results of the studies.

Abatacept, a T-cell inhibitor, was found as an independent risk factor for HBr in patients with rheumatic diseases. In a study, reverse seroconversion was seen in 6 patients (8.6%) treated with abatacept, and abatacept was also observed to be an independent risk factor as rituximab for HBr.^15^ In another study conducted on RA patients, reactivation was seen in 3 patients (10.3%). However, abatacept was not shown to be a risk factor for reactivation in multivariate analysis.^9^ We have 13 patients exposed to abatacept, and none of them had HBr. The number of abatacept-exposed patients or difference in etiology may be the reason for this difference from previous studies.

Tocilizumab is an anti-interleukin-6 agent, and tofacitinib is a Janus-kinase inhibitor that is used in IMID. In previous studies, HBr was seen in 1 patient among 64 HBsAg-negative and anti-HBc IgG-positive RA patients using tocilizumab.^7^ Also, 1 HBr was seen in 25 RA patients in another study.^9^ Besides this, there was no HBr with tocilizumab exposure in the other 2 studies.^8,17^ In that 2 studies, no HBr was seen with tofacitinib.^8,18^ In our cohort, 16 patients were exposed to tocilizumab and 10 patients were exposed to tofacitinib, and there was no HBr among these patients.

In the light of the current literature, there are no data about ustekinumab, secukinumab, and ixekizumab. In our study, we did not find HBr in 28 ustekinumab-, 15 secukinumab-, and 3 ixekizumab-exposed patients. Larger sample size and more studies are needed to prove the safety of these agents for HBr in inactive HBV patients.

Retrospective design is an important limitation of our study. Lacking HBV DNA monitoring in follow-up with a rate of 9.5% is another limitation. On the other hand, this is novel data for several biologic agents. Also, this study has a sufficient sample size to contribute current knowledge about this topic.

## Conclusions

In conclusion, prophylactic treatment and follow-up are not clear for HBsAg-negative and anti-HBc IgG-positive patients undergoing biologic treatment. In our study, we observed only 2 HBr in 221 HBsAg-negative and anti-HBc IgG-positive patients treated with biologic agents. We showed a low risk for HBr in HBsAg-negative and anti-HBc IgG-positive patients under biologic treatment. The prophylactic strategy could be reviewed with current literature to avoid unnecessary treatment costs and potential side effects.

## Figures and Tables

**Table 1.** Sociodemographic and Clinical Variables of Immune-Mediated Inflammatory Disease Patients

**Age** (mean ± SD)	54.08 ± 11.69 years
**Sex** (n)	
	Female	115 (52.0%)
Male	106 (48.0%)
**Diagnosis** (n)	
Rheumatoid arthritis	55 (24.9%)
Spondylarthritis	76 (34.4%)
Inflammatory bowel disease	20 (9.0%)
Psoriasis	57 (25.8%)
Behçet’s disease	7 (3.2%)
Takayasu arteritis	4 (1.8%)
Still disease	1 (0.5%)
Temporal arteritis	1 (0.5%)
**Total biologic exposure time** (median)	55 (range: 2-179) months
**Total number of the biologics exposure** (n)	1 (range: 1-6)
**Antiviral prophylaxis for hepatitis B** (n)	
	No	173 (78.3%)
Yes Tenofovir Entecavir Lamivudine	48 (21.7%) >
**Hepatitis B surface antibody (n)**	
	Positive	185 (83.7%)
Negative	36 (16.3%)
**Hepatitis B DNA screening prior to biologic exposure (n)**	
	Screened	56 (25.3%)
Not screened	165 (74.7%)
**Concomitant immunomodulatory agent exposure (n)**	
	No	69 (31.2%)
Yes Azathioprine Methotrexate Leflunomide Cyclosporine Methotrexate and cyclosporine Leflunomide, cyclosporine, and methotrexate	152 (68.8%) 22 (10.0%) 72 (32.6%) 23 (10.4%) 3 (1.4%) 31 (14.0%) 1 (0.5%)
**Concomitant corticosteroid exposure (n)**	
No	137 (62.0%)
Yes Exposure above 20 mg for more than 20 days Exposure below 20 mg or above 20 mg with an exposure of 20 days or less	84 (38.0%) 14 (6.3%) 70 (31.7%)

SD, standard deviation.

**Table 2.** Biologic Agent Exposure According to the Treatment Line

	**Biologic Agent**	**n (%)**	**Median Exposure Time (Months)**
	**First line **	Infliximab	41 (18.6)	42 (range: 5-149)
		Adalimumab	67 (30.3)	29 (range: 2-167)
		Etanercept	38 (17.2)	59 (range: 6-120)
		Certolizumab	13 (5.9)	10 (range: 3-52)
		Golimumab	14 (6.3)	49.5 (range: 10-86)
		Ustekinumab	17 (7.7)	17 (range: 5-61)
		Secukinumab	5 (2.3)	19 (range: 5-23)
		Abatacept	11 (5.0)	42 (range: 6-85)
		Ixekizumab	1 (0.5)	6 (range: 6-6)
		Tocilizumab	4 (1.8)	42 (range: 3-68)
		Tofacitinib	10 (4.5)	38 (range: 13-56)
**Second line**	Infliximab	13 (16.9)	12 (range: 1-69)
		Adalimumab	22 (28.6)	14 (range: 1-159)
		Etanercept	12 (15.6)	22.5 (range: 5-78)
		Certolizumab	7 (9.1)	48 (range: 4-59)
		Golimumab	3 (3.9)	82 (range: 46-84)
		Ustekinumab	7 (9.1)	31 (range: 5-64)
		Secukinumab	3 (3.9)	11 (range: 9-11)
		Abatacept	1 (1.3)	91 (range: 91-91)
		Ixekizumab	1 (1.3)	5 (range: 5-5)
		Tocilizumab	4 (5.2)	41 (range: 15-59)
		Risankizumab	4 (5.2)	31 (range:14-55)
**Third line**	İnfliksimab	3 (10.3)	16 (range: 11-66)
	Adalimumab	4 (13.8)	19 (range: 1-32)
	Etanersept	5 (17.2)	45 (range: 15-79)
	Sertolizumab	5 (17.2)	52 (range: 19-60)
	Golimumab	2 (6.8)	77 (range: 72-82)
	Ustekinumab	3 (10.3)	31 (range: 25-32)
	Sekinumab	4 (13.8)	11.5 (range: 8-22)
	Abatasept	1 (3.4)	15 (range: 15-15)
	İkzekizumab	1 (3.4)	8 (range: 8-8)
	Tocilizumab	1 (3.4)	24 (range: 24-24)
**Fourth line**	Sertolizumab	2 (20.0)	24.5 (range: 10-39)
	Golimumab	1 (10.0)	2 (range: 2-2)
	Ustekinumab	1 (10.0)	3 (range: 3-3)
	Sekinumab	1 (10.0)	17 (range: 17-17)
	Tocilizumab	1 (10.0)	11 (range: 11-11)
	Risankizumab	1 (10.0)	12 (range: 12-12)
**Fifth line**	Sertolizumab	1 (10.0)	15 (range: 15-15)
	Sekinumab	2 (20.0)	11.5 (range: 11-12)
**Sixth line**	Sekinumab	1 (100.0)	30 (range: 30-)
